# *BRAF* mutation testing with a rapid, fully integrated molecular diagnostics system

**DOI:** 10.18632/oncotarget.4723

**Published:** 2015-07-27

**Authors:** Filip Janku, Bart Claes, Helen J. Huang, Gerald S. Falchook, Benoit Devogelaere, Mark Kockx, Isabelle Vanden Bempt, Martin Reijans, Aung Naing, Siqing Fu, Sarina A. Piha-Paul, David S. Hong, Veronica R. Holley, Apostolia M. Tsimberidou, Vanda M. Stepanek, Sapna P. Patel, E. Scott Kopetz, Vivek Subbiah, Jennifer J. Wheler, Ralph G. Zinner, Daniel D. Karp, Rajyalakshmi Luthra, Sinchita Roy-Chowdhuri, Erwin Sablon, Funda Meric-Bernstam, Geert Maertens, Razelle Kurzrock

**Affiliations:** ^1^ Department of Investigational Cancer Therapeutics (Phase I Clinical Trials Program), The University of Texas MD Anderson Cancer Center, Houston, TX 77030, USA; ^2^ Biocartis NV, 2800 Mechelen, Belgium; ^3^ Sarah Cannon Research Institute at HealthONE, Denver, CO 80218, USA; ^4^ Department of Melanoma Medical Oncology, The University of Texas MD Anderson Cancer Center, Houston, TX 77030, USA; ^5^ Department of Gastrointestinal Medical Oncology, The University of Texas MD Anderson Cancer Center, Houston, TX 77030, USA; ^6^ Molecular Diagnostics Laboratory, The University of Texas MD Anderson Cancer Center, Houston, TX 77030, USA; ^7^ Department of Pathology, The University of Texas MD Anderson Cancer Center, Houston, TX 77030, USA; ^8^ Center for Personalized Cancer Therapy, Moores Cancer Center, The University of California San Diego, La Jolla, CA 92093, USA; ^9^ Cartagenia, 3001 Leuven, Belgium; ^10^ HistoGeneX NV, 2600 Berchem, Belgium

**Keywords:** BRAF, rapid, integrated, qPCR

## Abstract

Fast and accurate diagnostic systems are needed for further implementation of precision therapy of *BRAF-*mutant and other cancers. The novel Idylla^TM^ *BRAF* Mutation Test has high sensitivity and shorter turnaround times compared to other methods. We used Idylla to detect *BRAF* V600 mutations in archived formalin-fixed paraffin-embedded (FFPE) tumor samples and compared these results with those obtained using the cobas 4800 *BRAF* V600 Mutation Test or MiSeq deep sequencing system and with those obtained by a Clinical Laboratory Improvement Amendments (CLIA)-certified laboratory employing polymerase chain reaction–based sequencing, mass spectrometric detection, or next-generation sequencing. In one set of 60 FFPE tumor samples (15 with *BRAF* mutations per Idylla), the Idylla and cobas results had an agreement of 97%. Idylla detected *BRAF* V600 mutations in two additional samples. The Idylla and MiSeq results had 100% concordance. In a separate set of 100 FFPE tumor samples (64 with *BRAF* mutation per Idylla), the Idylla and CLIA-certified laboratory results demonstrated an agreement of 96% even though the tests were not performed simultaneously and different FFPE blocks had to be used for 9 cases. The Idylla^TM^ *BRAF* Mutation Test produced results quickly (sample to results time was about 90 minutes with about 2 minutes of hands on time) and the closed nature of the cartridge eliminates the risk of PCR contamination. In conclusion, our observations demonstrate that the Idylla test is rapid and has high concordance with other routinely used but more complex *BRAF* mutation–detecting tests.

## INTRODUCTION

With the identification of druggable molecular aberrations in cancer, we have increased our understanding of cancer biology and identified novel molecular targets for cancer therapy. [1–6] In particular, the identification of the *BRAF* V600 mutation hotspot in melanoma and other malignancies has led to the development of small-molecule kinase inhibitors targeting the *BRAF* oncogene. These *BRAF* inhibitors have revolutionized therapy for patients with *BRAF* V600–mutant advanced melanoma and demonstrated promising results in patients with other *BRAF* mutation–harboring diseases, including histiocytosis, hairy cell leukemia, non–small cell lung cancer, and biliary cancer. [4, 7–10]

*BRAF* inhibitors are contraindicated in the absence of a *BRAF* mutation. To determine whether a disease harbors a *BRAF* mutation, and thus whether treatment with *BRAF* inhibitors is appropriate, various methods to detect *BRAF* mutations in archived formalin-fixed, paraffin-embedded (FFPE) tissue samples are used. However, the methods that are currently routinely used for this purpose often require several hours to perform owing to time-consuming steps that include incubation, pipetting, and other processes. [11] In addition, for these molecular testing methods to be cost-effective, they are often performed on batches of samples, rather than single samples, thereby further increasing the turnaround time from sample to result. Consequently, results from these tests may not be available for at least several days or even weeks. [12] Such delays can hinder the delivery of effective treatment and thus have negative implications for care, especially in patients with a rapidly progressing disease such as advanced melanoma. [13]

A faster and simplified method for detecting *BRAF* mutations in FFPE tumor samples is the novel, fully integrated, real-time polymerase chain reaction (PCR)-based Idylla^TM^ system. Unlike current routinely used technologies for *BRAF* mutation detection, the Idylla^TM^ *BRAF* Mutation Test does not require manual sample preprocessing steps such as deparaffinization, FFPE tissue digestion, or DNA extraction because all these steps are integrated within a single-use cartridge. A complete FFPE tissue section or macrodissected FFPE material is placed directly into the cartridge and is subsequently processed by the Idylla^TM^ system, which provides automated sample processing, real-time PCR–based mutation detection, and result reporting. In the present study, we compared the performance of the Idylla^TM^ *BRAF* Mutation Test with that of other routinely used diagnostic methods for detecting *BRAF* V600 mutations.

## RESULTS

### Analytical sensitivity and specificity

**Figure 1:**
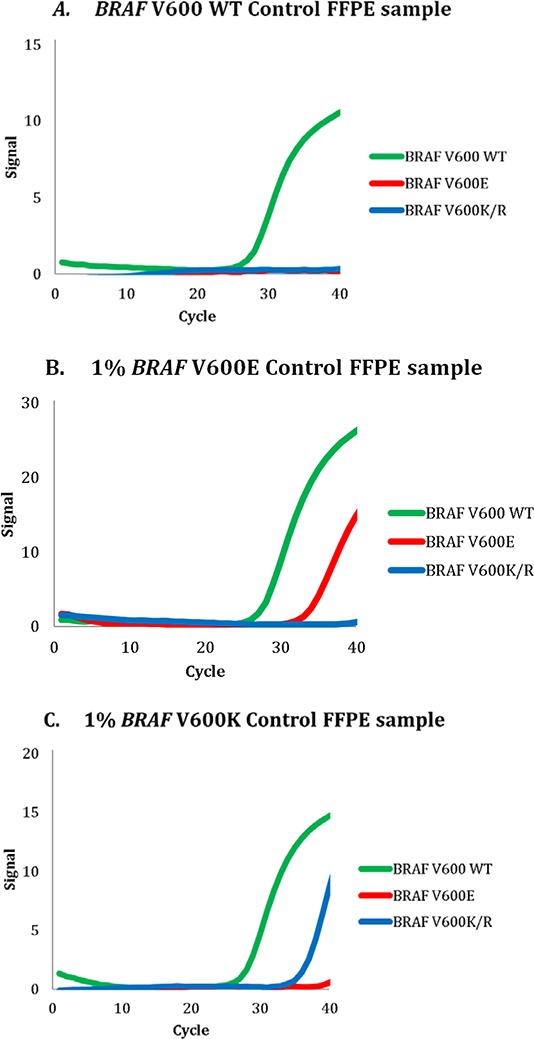
Representative examples of polymerase chain reaction curves for formalin-fixed paraffin-embedded cell line mixtures containing **A.** wild-type *BRAF*, **B.** 1% *BRAF* V600E, or **C.** 1% *BRAF* V600K. WT, wild type.

To determine the sensitivity of the Idylla^TM^ *BRAF* Mutation Test (hereafter referred to as Idylla) in detecting *BRAF* V600 mutations in FFPE material, we used the test to analyze eight different sections of commercially available FFPE cell-line blends containing wild-type *BRAF* only, 1% *BRAF* V600E or 1% *BRAF* V600K in a wild-type *BRAF* background and demonstrated 100% agreement among eight independent experiments for each respective mutation (Table 1, Figure 1A–C).

**Table 1:** Performance of the Idylla^TM^ *BRAF* Mutation Test on control formalin-fixed, paraffin-embedded samples containing 1% or no *BRAF* V600 mutation

Control sample	V600E mutation detected	V600K mutation detected	No mutation detected
1% *BRAF* V600E	8/8	0/8	0/8
1% *BRAF* V600K	0/8	8/8	0/8
*BRAF* V600 wild-type	0/8	0/8	8/8

To assess the reproducibility of the Idylla ^TM^*BRAF* V600 Mutation Test across different Idylla^TM^ instruments and among different operators, three operators used the test to repeatedly analyze consecutive sections from the same control 1% *BRAF* V600K FFPE sample 139 times on 7 different instruments using cartridges from two different production batches. *BRAF* V600K mutations were identified in 100% of the sections, indicating the high reproducibility of the test's results, even in cases with low abundant mutations, regardless of instrument and operator variation (Supplementary Figure S1).

To determine the specificity of the Idylla^TM^ *BRAF* Mutation Test in detecting *BRAF* V600 mutations, we used the test to analyze a high number of wild-type *BRAF* genomic DNA copies (8 × 10^4^ per PCR reaction) from the *BRAF* wild-type CHL-1 cell line to identify the extent of cross-reactivity between the *BRAF* wild-type allele and the *BRAF* V600E and V600K reactions. Unlike the DNA recovered from FFPE samples, which is degraded by the formalin fixation and only partially amplifiable by PCR, the unfixed, high-quality genomic DNA recovered from the CHL-1 cell line enabled us to maximally challenge the specificity of the assay. In this experiment, the delta Ct between the specific signal of the *BRAF* V600 wild-type reaction and the cross-reactivity signal in the *BRAF* V600E and *BRAF* V600K reactions was greater than 20, demonstrating that the mutation detection reactions are highly specific even in the presence of a high number of wild-type alleles (Supplementary Figure S2).

### Performance of Idylla versus cobas and MiSeq

We initially tested the Idylla^TM^ *BRAF* Mutation Test (hereafter referred to as Idylla) capacity to detect *BRAF* V600 mutations using 73 unprocessed (i.e., without macro- or micro dissection) FFPE tumor samples from patients with diverse cancers (melanoma, *n* = 30; colorectal cancer, *n* = 23; non-small cell lung cancer [NSCLC], *n* = 12; papillary thyroid cancer, *n* = 5; breast cancer, *n* = 3) obtained from commercial suppliers as outlined in Methods section (Table 2). No samples produced an invalid result. Idylla detected *BRAF* V600 mutations in 12 of 30 melanomas (40%), 1 of 23 colorectal cancers (4%), and 2 of 5 papillary thyroid cancers (40%) but did not detect *BRAF* V600 mutations in breast cancers or NSCLC.

**Table 2:** *BRAF* V600 mutations in different tumor types detected by Idylla

Tumor type (n)	*BRAF* V600E	*BRAF* V600K/R	*BRAF* V600 wild-type
Melanoma (30)	9	3	18
Colorectal cancer (23)	1	0	22
Lung cancer (12)	0	0	12
Thyroid cancer (5)	2	0	3
Breast cancer (3)	0	0	3

From the 73 FFPE tumor samples, we randomly selected 45 samples with wild-type *BRAF* and all 15 samples with *BRAF* V600 mutations as per Idylla and subjected these 60 samples to the cobas *BRAF* V600 Mutation Test, a U.S. Food and Drug Administration–approved companion diagnostic, according to manufacturer's instructions. The sample size of 60 samples was expected to be adequate to demonstrate concordance of 95%–100% (kappa 0.9, 95% confidence interval [CI] +/− 0.15). To help maximize the sensitivity of the cobas test, we used macrodissection to enrich the tumor area of 42 of the 60 tumor samples (70%). Of these 60 FFPE tumor samples, 2 (1 melanoma and 1 breast cancer) could not be analyzed with cobas owing to insufficient DNA concentrations. For 56 of the remaining 58 samples (97%), the Idylla and cobas results were in overall agreement (kappa 0.91, standard error [SE], 0.07, 95% CI, 0.78–1.00). Compared with Idylla, cobas had a sensitivity of 87% (95% CI, 0.60–0.98), specificity of 100% (95% CI, 0.92–1.00), positive predictive value of 100% (95% CI, 0.75–1.00), and negative predictive value of 96% (95% CI, 0.85–0.99; Table 3). Of interest, the 2 samples in which Idylla but not cobas detected *BRAF* mutations (1 *BRAF* V600E mutation and 1 *BRAF* V600R mutation) contained less than 25% tumor cells. As described below, the Idylla results for both these samples were confirmed using an independent method.

**Table 3:** Concordance between Idylla and other methods in detecting *BRAF* V600 mutations in formalin-fixed paraffin-embedded (FFPE) tumor tissue samples

Concordance between cobas and Idylla testing of FFPE tumor tissue samples (*N* = 58)		
	*BRAF* mutation (cobas)	*BRAF* wild-type (cobas)
*BRAF* mutation (Idylla)	13	2
*BRAF* wild-type (Idylla)	0	43
Observed agreements	56 (97%); kappa, 0.91, SE, 0.07; 95% CI, 0.78–1.00	
**Concordance between MiSeq and Idylla testing of FFPE tumor tissue samples (*N* = 58)**		
	*BRAF* mutation (MiSeq)	*BRAF* wild-type (MiSeq)
*BRAF* mutation (Idylla)	15	0
*BRAF* wild-type (Idylla)	0	43
Observed agreements	58 (100%); kappa, 1.00, SE, 0.00; 95% CI, 1.00–1.00	
**Concordance between laboratory and Idylla testing of FFPE tumor tissue samples (*N* = 100)**		
	*BRAF* mutation (CLIA)	*BRAF* wild-type (CLIA)
*BRAF* mutation (Idylla)	61	1
*BRAF* wild-type (Idylla)	3	35
Observed agreements	96 (96%); kappa, 0.91, SE, 0.04; 95% CI, 0.83–1.00	
Sensitivity	95% (95% CI, 0.87–0.99)	
Specificity	97% (95% CI, 0.85–1.00)	
Positive predictive value	98% (95% CI, 0.91–1.00)	
Negative predictive value	92% (95% CI, 0.79–0.98)	

Abbreviations: SE, standard error; CI, confidence interval.

To validate the results obtained with Idylla and cobas, we subjected the 60 FFPE tumor samples (45 with wild-type *BRAF* and 15 with *BRAF* V600 mutations as per Idylla) to mutation analysis using the MiSeq deep sequencing system, in which the *BRAF* V600–surrounding region was amplified by PCR and sequenced at high depth (coverage >5000x) to facilitate the identification of mutations present in at least 1% of the DNA. Of these 60 samples, 2 had insufficient coverage and were excluded from further analysis. For the remaining 58 samples, the Idylla and MiSeq results demonstrated an overall agreement of 100% (kappa 1.00, SE 0.00; 95% CI, 1.00–1.00). Compared with MiSeq, Idylla had a sensitivity of 100% (95% CI, 0.78–1.00), specificity of 100% (95% CI, 0.92–1.00), positive predictive value of 100% (95% CI, 0.78–1.00), and negative predictive value of 100% (95% CI, 0.92–1.00; Table 3). The MiSeq analysis also revealed that the two low-tumor-cellularity samples in which Idylla but not cobas had identified a *BRAF* mutation contained 4.6% V600E and 5.7% V600R, respectively, which is in line with the overall specifications of the cobas test.

### Independent clinical validation of the Idylla^TM^ *BRAF* mutation test

We then used Idylla in a retrospective study to test FFPE tumor samples from 100 patients with advanced cancers (melanoma, *n* = 38; colorectal cancer, *n* = 25; papillary thyroid carcinoma, *n* = 12; ovarian cancer, *n* = 5; cholangiocarcinoma, *n* = 3; head and neck cancer, *n* = 3; gastrointestinal stromal tumor, *n* = 2; NSCLC, *n* = 2; other, *n* = 10, Table 4) whose *BRAF* V600 mutation status had been previously determined in MD Anderson's Clinical Laboratory Improvement Amendments (CLIA)-certified Molecular Diagnostics Laboratory from routine clinical biopsies and resections. The CLIA laboratory detected *BRAF* V600 mutations in 66 of the 100 specimens (66%), and Idylla detected *BRAF* V600 mutations in 63 of the specimens (63%). The results of Idylla and the CLIA laboratory had overall agreement in 96 cases (96%; kappa, 0.91, SE, 0.04; 95% CI, 0.83–1.00). Compared with the CLIA laboratory, Idylla had a sensitivity of 95% (95% CI, 0.87–0.99), specificity of 97% (95% CI, 0.85–1.00), positive predictive value of 98% (95% CI, 0.91–1.00), and negative predictive value of 92% (95% CI, 0.79–0.98; Table 3). We always attempted to obtain the identical tissue blocks that were used by the CLIA laboratory for testing with Idylla; however, this was not possible in 9 cases. Of interest, of the 9 samples for which a different block was analyzed by the Idylla test, only 1 had discrepant results with the CLIA laboratory (*BRAF* V600K by CLIA, but wild-type by Idylla) resulting in an overall agreement of 89% for this subset (Supplementary Table S1). One (colorectal cancer with *BRAF* V600K mutation by the CLIA, but not Idylla) of four patients with discrepant *BRAF* V600 mutation status between the CLIA laboratory and Idylla received a MEK inhibitor with disease progression and one patient (melanoma with *BRAF* V600E mutation by the CLIA, but not Idylla) received a combination of BRAF inhibitor with chemotherapy and responded for 3 months. In addition, a patient with prostate carcinoma (*BRAF* V600E mutation by the CLIA, but not Idylla) and colorectal cancer (*BRAF* V600E mutation by the Idylla, but not CLIA) never received BRAF or MEK inhibitors.

**Table 4:** Tumor types of 100 patients with advanced cancers with known *BRAF* V600 status from the CLIA laboratory tested with Idylla

Tumor type	No. of patients	No. of *BRAF* V600 mutations detected (CLIA)	No. of *BRAF* V600 mutations detected (Idylla)
Melanoma	38	34	33
Colorectal cancer*	25	9	9
Papillary thyroid cancer	12	12	12
Ovarian cancer	5	1	1
Cholangiocarcinoma	3	2	2
Head and neck cancer	3	0	0
Sarcoma	3	0	0
Gastrointestinal stromal tumor	2	1	1
Non-small cell lung cancer	2	2	2
Esophageal cancer	1	0	0
Adrenocortical cancer	1	0	0
Prostate cancer	1	1	0
Appendiceal cancer	1	1	1
Neuroendocrine carcinoma of unknown primary	1	0	0
Glioblastoma	1	1	1
Thymoma	1	0	0

* One wild-type sample from CLIA showed *BRAF* V600 mutation on Idylla and one BRAF V600-mutant sample from CLIA demonstrated wild-type on Idylla

## DISCUSSION

The present study's findings demonstrate that the Idylla^TM^ *BRAF* Mutation Test can reproducibly detect *BRAF* V600 mutations in FFPE samples with as little as 1% mutant DNA in a wild-type background and that the test's performance is on par with that of other routinely used but more complex methods of *BRAF* mutation detection.

First, we found an overall agreement of 97% between Idylla and cobas on 60 FFPE tumor samples. Idylla detected *BRAF* V600 mutations (4.6% V600E and 5.7% V600R according to MiSeq) in two samples that had relatively low tumor cellularity and that cobas identified as having wild-type *BRAF*, plausibly due to the test's higher detection limit (at least 5% of V600E mutant DNA) and because cobas is not validated for the detection of V600R. Furthermore, the Idylla results were confirmed using the MiSeq next-generation sequencing platform with 100% concordance. In an independent study, we found an overall agreement of 96% between Idylla and a CLIA-certified laboratory on 100 FFPE samples despite the fact that the testing was not performed simultaneously and that a different block had to be used for Idylla testing in 9% of cases. Although intratumoral heterogeneity for the *BRAF* V600 mutation might confound the concordance analysis, this effect appears to have a limited impact in our study, since the overall agreement level between both tests reached 96%. [14]

The workflow complexities and turnaround times of the different *BRAF* mutation–detecting methods used in the present study differ considerably. Unlike the FFPE sample processing steps in methods such as cobas, the FFPE sample processing steps in Idylla are completely integrated within one cartridge, which eliminates the need for manually performing time-consuming procedures such as deparaffinization, tissue digestion, and DNA extraction. Both the cobas test and MiSeq platform require a separate upfront DNA extraction step, which can take up to 3 hours, including about 1 hour for manual pipetting and incubation; subsequent PCR testing requires an additional 2 hours, including 30 minutes for manual procedures, when cobas is used. While these procedures are mostly performed in a sample batching mode, Idylla allows random access analysis of individual samples, leading to a turnaround time of hours instead of up to several weeks. The MiSeq workflow is even more complex and time-consuming than that of cobas owing to the different steps needed for preparing the sequencing library and performing the sequencing itself. In addition, for both cobas and MiSeq, the use of separate pre-PCR and post-PCR rooms to prevent sample contamination is strongly recommended. In contrast, the Idylla^TM^ *BRAF* Mutation Test produces results in about 90 minutes with about 2 minutes of hands on time and the closed nature of the cartridge eliminates the risk of PCR contamination. Furthermore, given its simple workflow and quick turnaround time, the Idylla^TM^ system can be used at nearly any facility, including those that would not be able to implement technologies with more complex workflows such as cobas or MiSeq.

Although Idylla detects *BRAF* V600E, V600K, V600R, V600M, and V600D mutations, it does not distinguish between *BRAF* mutations occurring on the same nucleotide, i.e., the V600E/D or V600K/R/M mutations. However, this is clinically acceptable, because patients carrying either one of these mutations have been reported to benefit from treatment with a BRAF inhibitor.[15] Whereas the MiSeq platform can detect all of these mutations, the cobas test only detects *BRAF* V600E down to at least 5% of mutant allele and *BRAF* V600K down to at least 30% of mutant allele. In the present study, 70% of the samples analyzed with the cobas test were macrodissected to maximize the test's sensitivity; in contrast, no samples analyzed with the Idylla test were macrodissected, although the test is compatible with micro- or macrodissected tissue. In addition, the Idylla test detected a *BRAF* mutation in one unprocessed FFPE sample that MiSeq analysis revealed to have less than 5% *BRAF* mutation (i.e., 4.6% V600E), whereas the cobas test did not detect this mutation even in tissue that had been macrodissected. These findings suggest that Idylla can detect all clinically relevant *BRAF* V600 mutations and that, compared with the cobas test, Idylla has a higher sensitivity for detecting *BRAF V600* mutations and thus could be used to identify a greater number of patients who may benefit from treatment with a BRAF inhibitor.

In conclusion, the Idylla^TM^ *BRAF* Mutation Test, which offers an integrated and sensitive “sample-to-result” approach to detecting *BRAF* V600 mutations in FFPE samples, has a high concordance with routinely used methods for detecting *BRAF* V600 mutations in such samples.

## MATERIALS AND METHODS

### Tumor samples

From May 2012 until April 2014, patients with advanced cancers referred to the Department of Investigational Cancer Therapeutics at MD Anderson Cancer Center were enrolled in the study. FFPE tumor tissue samples from these patients were tested for *BRAF* V600 mutations in MD Anderson's CLIA-accredited Molecular Diagnostics Laboratory. Patients had to have enough archived tissue to allow the study and patient accrual was enriched to ensure that at least 60% of patients in the study had *BRAF* V600 mutations. Patient registration in the database, tumor pathology assessment, and tumor mutation analysis were performed at MD Anderson. The study was conducted in accordance with MD Anderson's Institutional Review Board guidelines. Additional FFPE tumor tissue samples from cancer patients who had signed an Institutional Review Board– or Ethical Committee–approved informed consent form were acquired through commercial suppliers (OriGene Technologies, Inc., Rockville, MD; Asterand Europe, Royston, Hertfordshire, United Kingdom).

### *BRAF* V600 mutation detection with the Idylla^TM^ BRAF mutation test

The Idylla^TM^ system (Biocartis, Mechelen, Belgium) is a random-access molecular diagnostic system that provides quantitative allele-specific real-time PCR–based sample-to-result functionality using a disposable cartridge that can detect and quantify up to 30 molecular biomarker groups from a variety of solid and liquid samples, including plasma and FFPE tissue. The instrument is composed of a sample preparation module integrated with a combined PCR thermocycling and fluorescence detection module. For FFPE specimens, the sample preparation module uses high intensity focused ultrasound technology to emulsify the paraffin and simultaneously rehydrate the tissue sample in an aqueous solution, thereby liberating DNA. Nucleic acids are then transported via microfluidic channels in the cartridge into 5 separate PCR chambers that contain pre-deposited dried PCR reagents (i.e., primers, probes, and enzymes). Each PCR chamber allows for the identification of up to 6 different biomarker groups (30 biomarker groups total), each of which can be composed of multiple individual biomarkers.

The Idylla *BRAF* Mutation Test (Biocartis, Mechelen, Belgium) is a single-use cartridge-based test designed to detect the nucleotide G1798 > A and T1799 > A changes in the *BRAF* gene with a sensitivity limit of 1% *BRAF* mutant DNA in wild-type background. The G1798 > A change is present in patients with V600K, V600R, and V600M mutations, whereas the T1799 > A change is present in patients with V600E, V600K, V600E2, and V600D mutations. The test requires an analytic time of about 90 minutes and a hands-on time of about 2 minutes. Once the sample is inserted into the cartridge and the lid is closed, the cartridge is sealed, thereby eliminating the possibility of cross-contamination between different samples. The test does not require that FFPE samples be manually deparaffinized or preprocessed; all reagents required for sample preparation (i.e., liberation of DNA from the FFPE section) and real-time PCR detection are included in the Idylla^TM^ cartridge. Although the test is compatible with macrodissected FFPE material, unprocessed FFPE sections were used in this study. For the clinical concordance analyses in the present study, a single 10-μm section per FFPE sample was processed according to the manufacturer's instructions by a molecular biology-trained operator who was blinded for the results of the reference methods.

### *BRAF* V600 mutation detection with the cobas 4800 BRAF V600 mutation test

The cobas 4800 *BRAF* V600 mutation test (Roche Molecular Systems, Pleasonton, CA) was performed according to the manufacturer's instructions at the College of American Pathologists– and CLIA-accredited molecular laboratory of HistoGeneX (Antwerp, Belgium). Briefly, 5-μm sections were stained with hematoxylin and eosin and examined by a certified pathologist who delineated the tumor areas and determined the tumor cell content. For optimal sensitivity, macrodissection was used to enrich the tumor area. DNA from two 5-μm sections was isolated using the cobas DNA Sample Preparation Kit (Roche Molecular Systems, Pleasonton, CA), diluted to 5 ng/μl, and tested on a cobas 4800 System v2.0 (Roche Molecular Systems) according to the manufacturer's instructions by a molecular biology-trained operator who was blinded for the Idylla^TM^ and MiSeq results.

### *BRAF* V600 mutation detection with the MiSeq deep sequencing system

For *BRAF* V600 mutation detection with the MiSeq deep sequencing system (Illumina, San Diego, CA), DNA was first extracted from 10-μm FFPE sections using a QIAamp FFPE tissue DNA extraction kit (Qiagen, Hilden, Germany) according to the manufacturer's instructions and eluted in 50 μl of elution buffer. Different samples' DNA was normalized to 10 ng/μl based on measurement by Nanodrop (Thermo Fisher Scientific, USA), and an amplicon surrounding the BRAF V600 codon was generated using the forward primer 5′-CTACTGTTTTCCTTTACTTACTACACCTCAGA-3′ and the reverse primer 5′-ATCCAGACAACTGTTCAA ACTGATG-3′. The DNA samples were combined with a PCR reaction mixture consisting of 10 mM Tris, 50 mM KCl, 500 μM each PCR primer, 3 mM MgCl_2_, 0.2 mM dNTPs, 2 U FastStart Taq DNA polymerase (Roche Diagnostics, Rotkreuz, Switzerland), and 2 μl DNA input. The mixture was then subjected to PCR thermocycling for 10 minutes at 95°C followed by 50 cycles of denaturation at 95°C for 10 seconds, annealing at 62°C for 15 seconds, an extension step of 1 minute at 72°C, and a final extension step of 7 minutes at 72°C. The PCR products were visualized on Experion gel to confirm the presence of a single band of the correct length. Subsequent steps, including PCR product purification, sample barcoding, preparation of the MiSeq sequencing library, MiSeq sequencing, and bioinformatics data analysis, were performed by the Nucleomics Core at the Vlaams Instituut voor Biotechnologie (Gent, Belgium). At least 5000x coverage of the target region in each sample was required for MiSeq to have sufficient sensitivity. During the data analysis, the mutation threshold percentage for variant reporting was set at 1% mutant allele in a wild-type background to identify all mutations occurring in at least 1% of the sample DNA.

### *BRAF* V600 mutation testing in MD Anderson's Molecular Diagnostics Laboratory

Archived tumor tissues obtained from enrolled patients' primary or metastatic sites using routine diagnostic and/or therapeutic procedures were subjected to mutation testing in the CLIA–certified Molecular Diagnostics Laboratory in the Division of Pathology and Laboratory Medicine at MD Anderson. All histologies were centrally reviewed at MD Anderson. DNA was extracted from microdissected paraffin-embedded tumor sections and analyzed using a PCR-based DNA sequencing method for *BRAF* V600 mutations utilizing primers designed by the Molecular Diagnostics Laboratory. In January 2011, the assay was changed to mass spectrometric detection (MassARRAY, Sequenom, San Diego, CA), and in March 2012, the assay was changed to next-generation sequencing (Ion Torrent, Life Technologies, Carlsbad, CA). The lower limit of detection is approximately 5–10%.

### Statistical analysis

Concordance among mutation analyses was assessed using the kappa coefficient, sensitivity, specificity, and positive and negative predictive values, which were calculated using the GraphPad software program (GraphPad Software, Inc.; La Jolla; CA).

## SUPPLEMENTARY FIGURES AND TABLE


