# Supplementary material for: BRAF mutation testing with a rapid, fully integrated molecular diagnostics system
Source: Oncotarget. 2015 Jul 27;6(29):26886–94. doi: 10.18632/oncotarget.4723 (PMC4694960; doi:10.18632/oncotarget.4723)
Supplement: Supplementary file 1 [file oncotarget-06-26886-s001.pdf]

SUPPLEMENTARY FIGURES AND TABLE

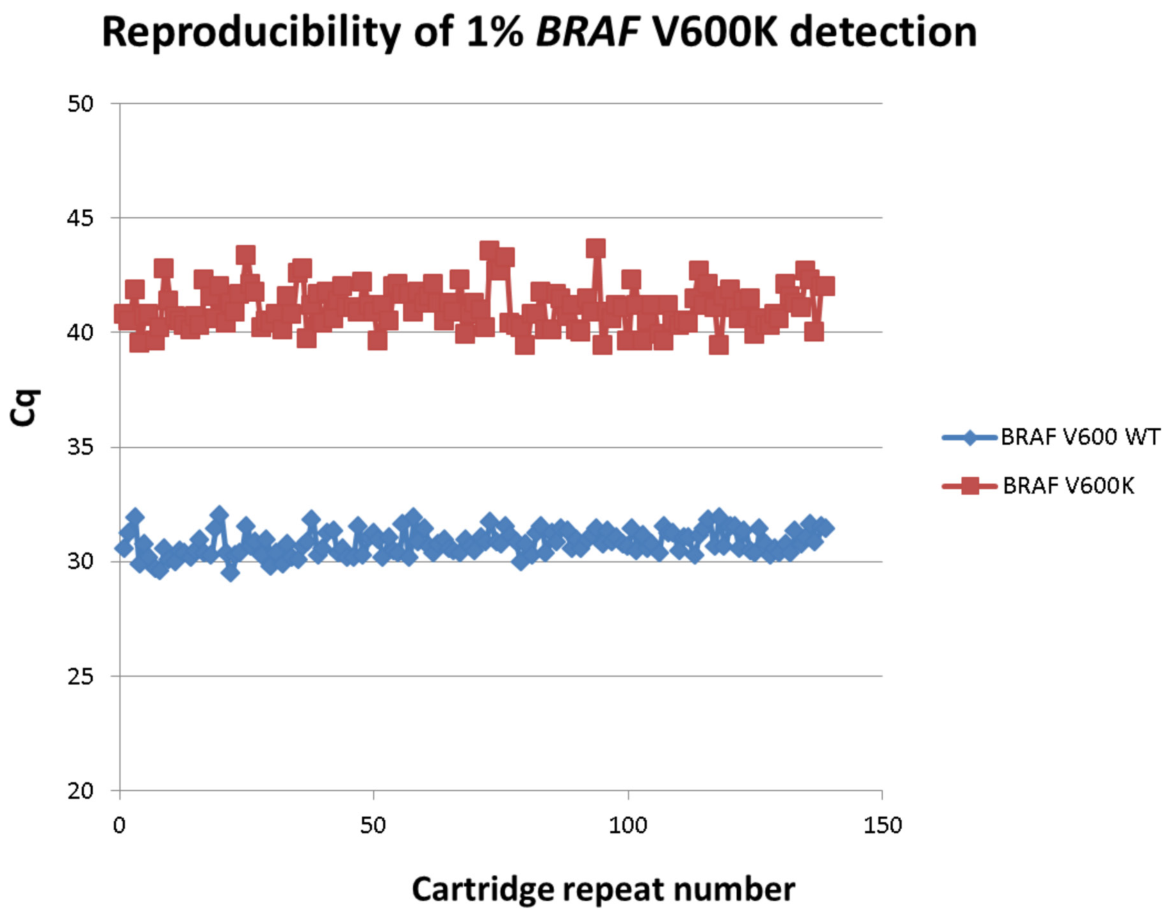

Supplementary Figure S1: To investigate the reproducibility of the Idylla *BRAF* mutation test across different Idylla instruments and operators, consecutive sections from one control FFPE sample containing 1% V600K were analyzed repeatedly for 139 times on 7 different instruments by 3 operators using cartridges from 2 different batches. One hundred percent of the samples were identified as *BRAF* V600K mutant, indicating the high reproducibility of the test regardless of instrument and operator variation, even for low abundant mutations. The treshold cycle or Cq value represents the cycle number at which the signal of the qPCR curve passes a certain baseline threshold. This number is used to estimate copy numbers present in the qPCR reaction.

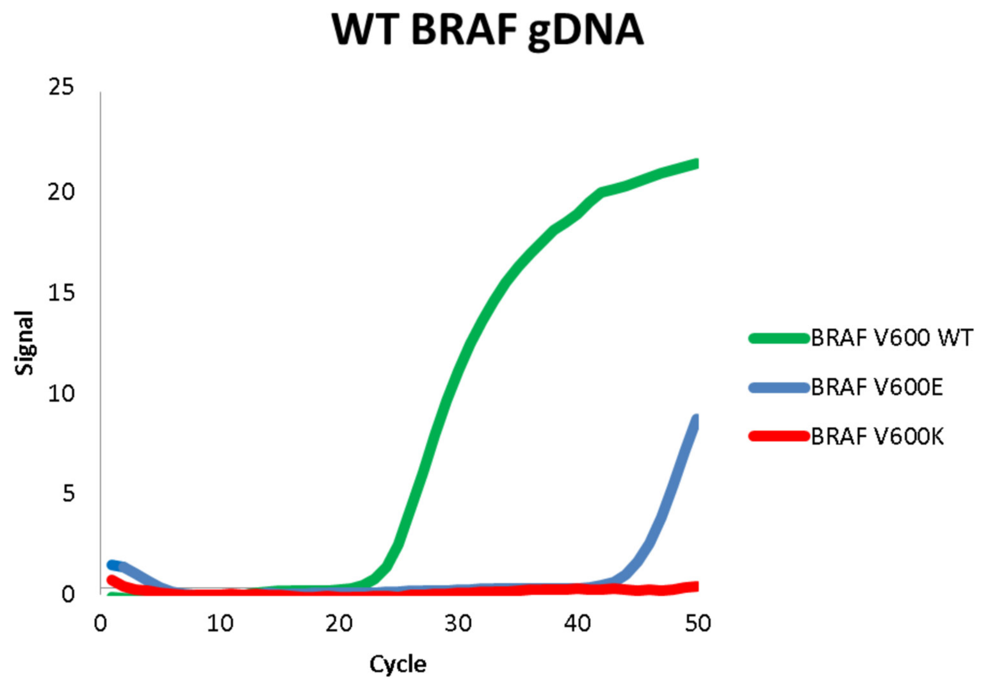

Supplementary Figure S2: Specificity analysis of the Idylla *BRAF* Mutation Test using a high amount of wild-type *BRAF* genomic DNA copies (8 x 10^4 per PCR reaction) from the *BRAF* wild-type CHL-1 cell line. The delta Ct (threshold cycle) between the specific signal of the BRAF V600 wild-type reaction (green) and the cross-reactivity signal in the V600E (blue) and V600K (red) reactions was greater than 20 cycles, demonstrating that the mutation detection reactions are highly specific even in the presence of a high wild-type allele background.

Supplementary Table S1: Concordance between Idylla and CLIA laboratory results for 9 formalin-fixed, paraffin-embedded samples for which different blocks were analyzed

	***BRAF* mutation (CLIA)**	***BRAF* wild-type (CLIA)**
BRAF mutation (Idylla)	4	0
BRAF wild-type (Idylla)	1	4
Observed agreements	8 (89%); kappa, 0.78, SE, 0.20; 95% CI, 0.39–1.00	

Abbreviations: SE, standard error, CI, confidence interval.
